# Aducanumab: Appropriate Use Recommendations Update

**DOI:** 10.14283/jpad.2022.34

**Published:** 2022

**Authors:** J. Cummings, G.D. Rabinovici, A. Atri, P. Aisen, L.G. Apostolova, S. Hendrix, M. Sabbagh, D. Selkoe, M. Weiner, S. Salloway

**Affiliations:** 1. Chambers-Grundy Center for Transformative Neuroscience, Department of Brain Health, School of Integrated Health Sciences, University of Nevada Las Vegas (UNLV), Las Vegas, NV, USA; 2. Memory and Aging Center, Department of Neurology, Weill Institute for Neurosciences and Department of Radiology and Biomedical Imaging; University of California, San Francisco, San Francisco, CA, USA; 3. Banner Sun Health Research Institute, Banner Health, Sun City, AZ; Center for Brain/Mind Medicine, Harvard Medical School, Boston, MA, USA; 4. Alzheimer’s Treatment Research Institute, University of Southern California, San Diego, CA, USA; 5. Department of Neurology, Radiology, Medical and Molecular Genetics, Indiana University School of Medicine, Indianapolis, Indiana, USA; 6. Pentara Corporation, Millcreek Utah, USA; 7. Barrow Neurological Institute, Dignity Health/St Joseph’s Hospital and Medical Center, Phoenix, Arizona, USA; 8. Ann Romney Center for Neurologic Diseases, Department of Neurology, Brigham and Women’s Hospital, Harvard Medical School, Boston, Massachusetts, USA; 9. Department of Radiology and Biomedical Imaging, Medicine, Psychiatry and Neurology, University of California San Francisco, San Francisco, CA, USA; 10. Butler Hospital and Warren Alpert Medical School of Brown University, Providence RI, USA

**Keywords:** Alzheimer’s disease, aducanumab, Aduhelm, appropriate use, titration, ARIA, amyloid imaging, MRI

## Abstract

Aducanumab (Aduhelm) is approved in the United States for the treatment of patients with mild cognitive impairment due to Alzheimer’s disease or mild AD dementia. Aducanumab Appropriate Use Recommendations (AURs) have been published and have helped guide best practices for use of aducanumab. As real-world use has occurred and more information has accrued, the AURs require refinement. We update the AURs to better inform appropriate patient selection and improve shared decision-making, safety monitoring, and risk mitigation in treated patients. Based on evolving experience we emphasize the importance of detecting past medical conditions that may predispose to amyloid related imaging abnormalities (ARIA) or may increase the likelihood of ARIA complications including autoimmune or inflammatory conditions, seizures, or disorders associated with extensive white matter pathology. The apolipoprotein E ε4 (APOE4) genotype is strongly associated with ARIA and exhibits a gene dose effect. We recommend that clinicians perform APOE genotyping to better inform patient care decisions, discussions regarding risk, and clinician vigilance concerning ARIA. As most ARIA occurs during the titration period of aducanumab, we suggest performing MRI before the 5th, 7th, 9th, and 12th infusions to improve detection. Uncommonly, ARIA may be recurrent or serious; we suggest additional parameters for treatment discontinuation taking these observations into account. It is important to continue to learn from the real-world use of aducanumab and the AURs will continue to evolve as new information becomes available. This AUR update does not address efficacy, price, or insurance coverage and is provided to assist clinicians to establish best practices for use of aducanumab in the treatment of patients with mild cognitive impairment and mild Alzheimer’s dementia.

Aducanumab (Aduhelm) is approved in the United States (US) for the treatment of mild cognitive impairment (MCI) due to Alzheimer’s disease (AD) and mild AD dementia (1). It was approved by the US Food and Drug Administration (FDA) using the accelerated approval mechanism available for drugs without definitively proven clinical benefit based on effects on a biomarker considered reasonably likely to predict clinical outcomes (2). Additional data are required from post-marketing studies to determine if continued approval is warranted. We previously provided Appropriate Use Recommendations (AURs) to assist clinicians in instituting best practices in the use of aducanumab including patient selection, treatment administration and monitoring, management of amyloid-related imaging abnormalities (ARIA), and how to discuss available information with patients and families (3). These AURs have been widely used (accessed online 18,000 times by the index date of March 8, 2022) since their introduction and have helped inform clinicians, hospitals, and health care systems about the use and management of aducanumab. As aducanumab has become more widely available to clinicians, information about how to implement the treatment and how to avoid or minimize complications is accruing. Recent publications as well as the experience of the members of the Alzheimer’s Disease and Related Disorders Therapeutics Working Group (ADRD TWG; comprised of the Expert Panel who developed the AURs and additional expert members) provide the basis for this update (4–7).

The AURs complement the Prescribing Information (PI) for aducanumab approved by FDA. The recommendations contained in the AURs fulfill the requirements of the PI; in some cases, recommendations are made for safety monitoring or practice adjustments that are more extensive than those described in the PI. The AURs describe the diagnosis of AD with confirmation of amyloid abnormalities (by amyloid positron emission tomography [PET] or measurement of relevant cerebrospinal fluid analytes); discuss cognitive tests and scales that can be used to identify patients with MCI/mild AD dementia; describe clinical circumstances that exclude the use of aducanumab; and emphasize shared decision making with patients and families to ensure their understanding of the available information on potential benefits and harms associated with aducanumab. The AURs provide guidance on use of magnetic resonance imaging (MRI) screening to identify findings that exclude the use of aducanumab, MRI monitoring for ARIA, and management of ARIA if detected. Many of the recommendations were derived from the study protocols of the EMERGE and ENGAGE studies (available on clincaltrials.gov), which fully describe the populations in which efficacy and safety were studied. There is no reported systematic experience with use of aducanumab in populations other than those included in clinical trials.

This AUR update builds on new information that further critically informs best practices for use of aducanumab (4–6). To be comprehensive, we reiterate some of the original recommendations, note where new information has become available, and describe where the recommendations have been modified (Table 1).

The AURs are intended to assist clinicians by providing practice recommendations, as well as important caveats and considerations for clinical use of aducanumab. The recommendations are not meant to replace the role of clinical judgement when caring for individual patients.

## Appropriate Patient

Patients being considered for treatment with aducanumab require a diagnosis of AD based on clinical assessment and confirmation of the presence of brain amyloid.

### Clinical Characterization

Patients appropriate for treatment with aducanumab should have the clinical syndrome of MCI due to AD or mild AD dementia (8, 9). Candidates for treatment require a thorough clinical assessment including review of the course of cognitive, functional, and behavioral changes; evaluation of the patient’s medical history and current medications to help exclude other causes or contributors to cognitive impairment; review of systems to determine if there are symptoms of other organ disorders; and laboratory studies that, at a minimum, include complete blood count, comprehensive metabolic panel, liver function tests, vitamin B12 level, thyroid stimulating hormone, erythrocyte sedimentation rate, and C-reactive protein to rule out the most common metabolic causes of cognitive decline and identify pre-existing inflammatory conditions. Neurological and medical examinations are needed to identify features inconsistent with AD (e.g., focal neurological signs, parkinsonism) or establish evidence of cardiovascular compromise or other medical conditions that can impair cognition and require further evaluation.

When reviewing the patient’s medical history, special attention should be devoted to identifying autoimmune and inflammatory conditions (such as polymyalgia rheumatica, giant cell arteritis, psoriatic arthritis, systemic lupus erythematosus, amyloid-related angiitis, and cerebral amyloid angiopathy with inflammation) (10, 11), detecting any history of seizures, and ascertaining conditions that may be associated with extensive white matter changes, including history of transient ischemic attack or cerebrovascular disease. This review may reveal conditions that suggest that a patient is not a good candidate for treatment with aducanumab. ARIA associated with aducanumab may be more likely in patients with underlying autoimmune or inflammatory conditions or extensive white matter changes. In rare cases, seizures have occurred in the context of ARIA, and patients with a recent history of seizures should not be treated with aducanumab. Bleeding disorders exclude consideration of the use of aducanumab.

Participants in the EMERGE and ENGAGE trials met criteria for MCI or mild AD dementia and had Mini Mental State Examination (MMSE) (12) scores of 24-30 and Clinical Dementia Rating (CDR) global scores of 0.5 (0.5 can be MCI or mild AD dementia). Patients in this stage of AD have limited decline in cognitive function and no or modest impairment in activities of daily living. A structured mental status examination or neuropsychological testing is needed to verify the presence of MCI or mild AD dementia in potential treatment candidates. Some widely used tools such as the MMSE (12) may be insensitive to the earliest changes of MCI; in these cases, more comprehensive assessment with a battery of neuropsychological testing will characterize cognitive decline noted by the patient or care partner. Use of brief tools such as the Montreal Cognitive Assessment (MoCA) (13) may be helpful in detecting cognitive impairment.

Review of the patient’s medication regimen is an important part of determining eligibility for treatment with aducanumab. Treatment with anticoagulants is a contraindication to use of aducanumab. Although treatment with platelet anti-aggregation agents such as aspirin at modest doses is not specifically contraindicated, the patient and care partner should be informed that these drugs might increase the risk of microhemorrhage or conversion of ARIA of the edema type (ARIA-E) to ARIA of the hemorrhagic type (ARIA-H). Many patients will be on drugs commonly used to treat AD including donepezil, rivastigmine, galantamine, or memantine. These can be continued and do not require dose adjustment.

### Baseline MRI and Amyloid Verification

MRI identifies patients with cerebrovascular disease that may predispose them to ARIA and may reveal alternate cause of dementia that require evaluation (e.g., subdural hematoma, hydrocephalus). Patients should be excluded from treatment if they have evidence of acute or subacute hemorrhage, a macrohemorrhage, cortical infarction larger than 1.5 cm, one lacunar infarction larger than 1.5 cm, more than four microhemorrhages, more than one area of superficial siderosis, or extensive white matter disease indicative of ischemic injury (Table 2). Substantial white matter pathology of concern includes those with irregular periventricular white matter hyperintensities extending into deep white matter and moderate or advanced confluent areas of deep white matter hyperintensities (14, 15). MRI sequences appropriate for this baseline assessment include T1, T2 or fluid attenuated inversion recovery (FLAIR), T2* gradient recalled echo (GRE) sequences or susceptibility weighted imaging (SWI), if available, and diffusion weighted imaging (DWI). When possible, MRI should be obtained with a 3T magnet and include SWI sequences to improve detection of microhemorrhages. While a brain MRI obtained within the past year may be acceptable if there have been no clinical changes since the scan was performed, it is preferable to obtain a brain MRI when initiating treatment or within 3-4 months of beginning treatment. No change has been made in these criteria from the previous Appropriate Use Recommendations(3). The safety of aducanumab has not been studied in patients who have any of these exclusion criteria and the accuracy with which they forecast increased risk is unknown.

Aducanumab is an anti-amyloid monoclonal antibody (mAb) and the presence of amyloid abnormalities should be verified for any patient being considered for treatment with this agent. A clinical diagnosis of MCI or dementia may not predict the presence of brain amyloid. Up to 50% of patients with MCI and 20-25% of those with dementia attributed to AD do not have amyloid plaques indicative of the presence of AD-type pathology (16–18). Amyloid PET is the imaging approach used to identify the presence of amyloid plaques. A visual read of the scan by an expert reader determines if the scan is negative or positive. There are several types of approved amyloid imaging ligands(19), and all can be used for this determination.

Amyloid pathology can also be established by assessment of Aβ levels or Aβ ratios in the cerebrospinal fluid (CSF): Aβ 42/40; Aβ 42/total tau; Aβ 42/phosphotau [p-tau])(20). These measures provide an alternative to amyloid PET. There is good correspondence between amyloid plaque burden as determined by amyloid PET and CSF amyloid abnormalities in the diagnosis of AD (21).

### Genetic Characterization

The ε4 allele of the apolipoprotein E (APOE4) haplotype is a risk factor for AD, both increasing the likelihood of developing AD and decreasing the age of onset (22). Most AD patients with proven brain amyloidosis (as shown by amyloid PET or CSF studies) have one or two copies of the APOE4 gene. There is a gene dose effect with the e4 allele; 1 copy increases the risk of AD by 2-3-fold and having two copies increases the risk by up to 12 fold (23). Approximately 50% of patients with biomarker-confirmed AD are APOE4 heterozygotes and 20% are APOE4 homozygotes; in the EMERGE and ENGAGE trials, 51% of participants were heterozygotes, 16% were homozygotes and 33% were noncarriers. APOE4 interacts with amyloid to decrease clearance from the brain and increase amyloid aggregation and deposition, and APOE4 is implicated in non-amyloid dependent pathways including effects on the tau protein, tau-mediated neurodegeneration, alpha-synuclein, TAR DNA binding protein 43 (TDP-43), and microglia (24, 25). Amyloid is deposited in the cerebral vessels in AD; the APOE4 gene increases vascular amyloid and the presence of amyloid angiopathy in AD (26).

The APOE4 haplotype is associated with increased risk of ARIA in individuals treated with plaque-lowering mAbs (7). In combined data from the EMERGE and ENGAGE clinical trials, 20.3% of APOE4 noncarriers and 43% of APOE4 carriers developed ARIA-E (27). There was a dose effect with 35.9% of those who were heterozygous for the APOE4 (1 copy of the APOE4 haplotype) and 66% of homozygous individuals (with two copies of the haplotype) exhibiting ARIA (4, 7). Severe ARIA-E was observed in 11% of homozygotes, 4% of heterozygotes, and 2% of noncarriers. The APOE4 gene was also associated with ARIA-H including microhemorrhage (22.7% carriers; 12.4% noncarriers) and superficial siderosis (19.1%; 6.2%). In total, 8.2% of APOE4 carriers compared to 2.5% of noncarriers discontinued trial participation because of ARIA (4). Hospitalization for ARIA was uncommon, occurring in 1% of APOE4 carriers and 2% of noncarriers. These data and accumulating real-world experience indicate that individuals who are APOE4 homozygous are at greater risk of ARIA-E occurrence and may have a higher likelihood for ARIA-E recurrence, ARIA-E severity, and ARIA-E-related serious adverse events (4, 5).

Most patients (74%) with ARIA in the aducanumab Phase 3 trials had no associated symptoms, and ARIA was known only through detection with MRI(4). Of the 26% with symptoms, 67.7% were mild, 28.3% were moderate, and 4% were severe. Most ARIA resolves without treatment discontinuation; 98.2% of ARIA occurring in the participants receiving 10 mg/kg of aducanumab in the EMERGE and ENGAGE trials resolved. The overall rate of serious adverse events due to ARIA in the EMERGE and ENGAGE trials was 0.3%. Adherence to a treatment protocol comparable to that of EMERGE and ENGAGE is expected to result in a similarly low rate of complications. The most common symptoms in those with symptomatic ARIA were headache (46.6% of those with symptoms), confusion and mental status changes (14.6%), dizziness and vertigo (10.7%), and nausea or vomiting (7.8% )(Table 3). Fatigue, visual impairment, blurred vision, and gait disturbances occurred in a few patients. Seizures were reported in 0.4% of patients treated with 10 mg/kg of aducanumab.

APOE4 genotype testing is warranted for informed use of aducanumab. This represents a change from previous clinical practice recommendations to forego genotyping in the absence of a related therapy (28). Genotype data are now actionable. The risk of symptomatic ARIA in the EMERGE and ENGAGE trials was low, and it was uncommon for ARIA to be severely symptomatic. However, there have been reports from open label extension studies with aducanumab and patients treated in clinical practice of a small number of patients with severe ARIA that required hospitalization. These have occurred in APOE4 carriers and primarily in individuals homozygous for APOE4. The higher risk of ARIA in APOE4 carriers supports APOE genotyping of individuals being considered for treatment with aducanumab. This information will improve risk assessment and enhance communication of risk to patients and care partners. Patients express a desire to know the results of genetic tests if the information is medically actionable (29). Revealing genotype status including those genotypes relevant to chronic diseases such as AD has not resulted in enduring psychological harm (29, 30). APOE genotype results have implications for biologically related family members that should be discussed with the patient and family prior to obtaining the genotype. The increased emphasis on the importance of genetic testing and its expanding clinical role is one of the real-world learnings from more widespread use of aducanumab and is a change from our previous AURs(3).

## Appropriate Administration and Monitoring of Aducanumab Therapy

**Figure 1.**
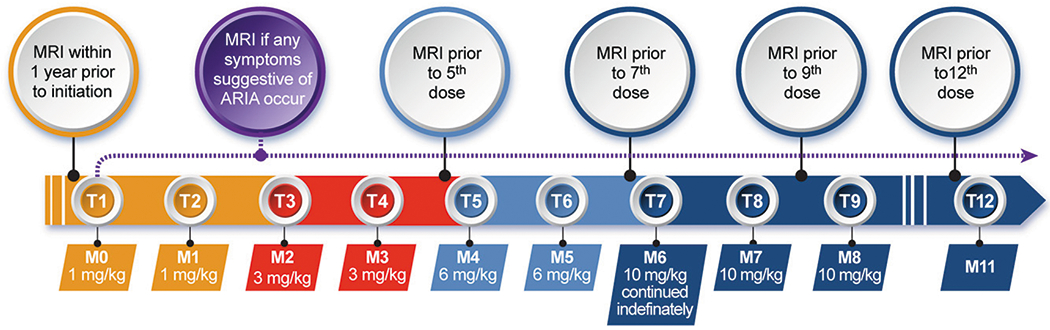
Schedule for aducanumab dosing and routine MRIs to monitor for the possible occurrence of ARIA

Aducanumab uses weight-adjusted dosing. The target dose is 10 mg/kg and is reached by titrating at two-month intervals from the starting dose of 1 mg/kg to 3 mg/kg, then to 6 mg/kg, and to then to the highest dose of 10 mg/kg as recommended in the PI. Infusions 1 and 2 administer 1 mg/kg, infusions 3 and 4 provide 3 mg/kg, infusions 5 and 6 dispense 6 mg mg/kg, and the 10 mg/kg dose begins with infusion 7 and is the dose to be continued (Figure 1). Titration or continuation of therapy may be suspended for acute illnesses --- myocardial infarction, acute conditions such as pneumonia, cancer therapy --- based on clinician judgement and patient/care partner preferences. Aducanumab is administered intravenously in saline solution and requires about 1 hour to infuse. Infusion reactions are very rare.

ARIA can occur at any time after treatment is initiated and vigilance for ARIA is required for all patients receiving aducanumab. Appropriate MRI sequences to detect ARIA-E and ARIA-H include FLAIR, T2*-GRE, and SWI (31). In EMERGE and ENGAGE most (72.7%) ARIA events occured during the titration phase and in the span of the first 8 doses (4). ARIA monitoring includes both routine monitoring with MRI and out-of-sequence symptom-based assessment if signs or symptoms suggestive of ARIA occur. In view of the emerging information that ARIA is most likely to occur before the 10 mg/kg dose is reached, the AUR update proposes that MRIs be obtained routinely before the 5th, 7th, 9th, and 12th doses (Figure 1). The proposed schedule reflects the imaging protocol used in the EMERGE and ENGAGE trials, and adoption of this more conservative schedule is warranted until more information on the community use of aducanumab is available. This added vigilance is to ensure detection of ARIA events early during aducanumab initiation when they are most likely to occur. MRI monitoring is especially important for APOE4 homozygotes who are at increased risk for ARIA. MRI monitoring information may evolve as more experience is gained with aducanumab. Genotype-specific monitoring based on APOE carrier status is a possible alternative.

**Figure 2.**
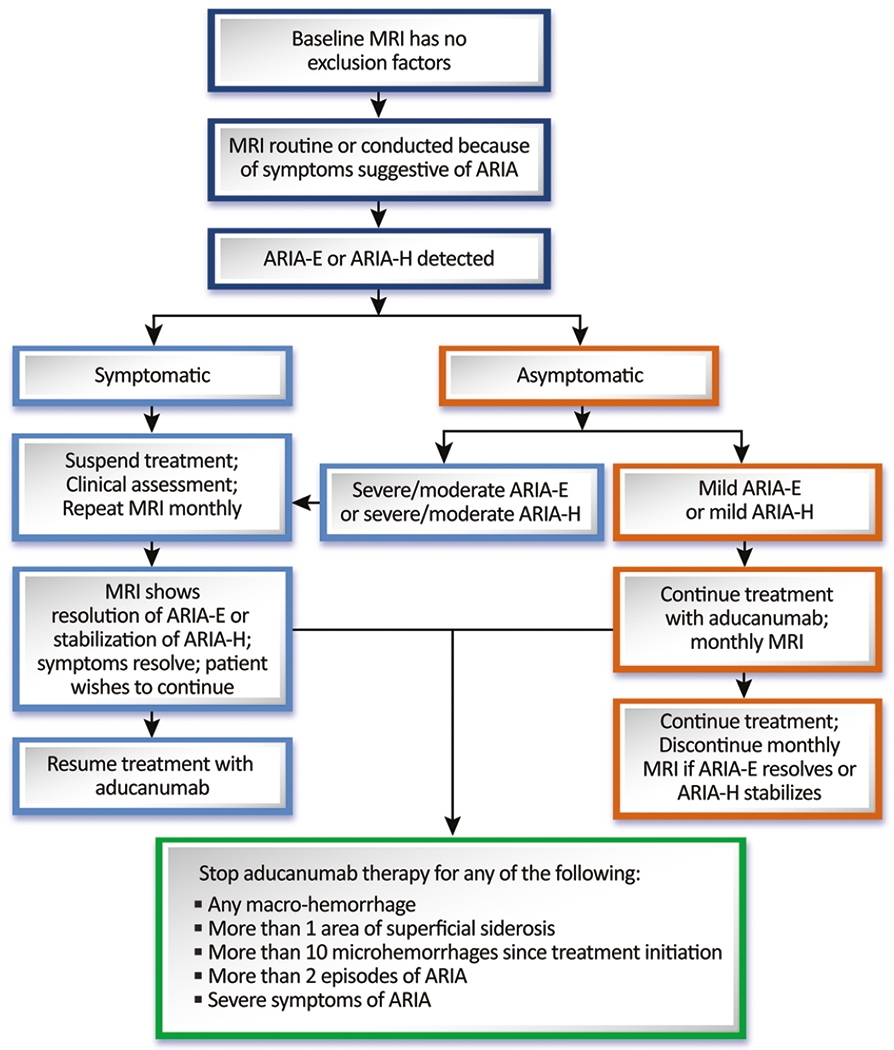
Management strategy for detecting and managing ARIA

If ARIA is detected, specific management strategies are indicated. If ARIA is of mild radiographic severity, dosing can continue with monthly MRI to detect any worsening. If the asymptomatic ARIA is moderate or severe or if the mild ARIA progresses to become moderate or severe, we recommend that dosing be interrupted, and MRI repeated monthly. Treatment (at the same dose the patient was receiving when the dosing was postponed) may be re-initiated once ARIA-E resolves or ARIA-H stabilizes. If ARIA is symptomatic, treatment is interrupted until monthly MRIs show that the ARIA-E has resolved, or the ARIA-H has stabilized (Figure 2).

In the setting of the EMERGE and ENGAGE trials, steroids were the most used agents to attempt to reduce swelling observed with ARIA-E or ARIA-H. Treatments used included methylprednisolone, prednisone, and dexamethasone. In the most severe symptomatic cases, high-dose glucocorticoid therapy can be considered; a prudent regimen is methylprednisolone 1 gm intravenously per day for 5 days followed by oral prednisone, 60 mg per day, slowly tapered over weeks. Some recent cases of ARIA were associated with seizures or status epilepticus. Electroencephalography should be performed to detect epileptiform activity and treatment with an anticonvulsant (e.g., levetiracetam) instituted promptly if evidence of seizure activity is detected. Careful clinical evaluation and monitoring should be performed until the ARIA and any related symptoms resolve. This management approach is more specific in the use of steroids and anticonvulsants for the management of severe ARIA and associated complications compared to the previous version of the AURs (3).

In the EMERGE and ENGAGE trials, 10.6% of patients who received 10 mg/kg had recurrent ARIA(7). The risk of recurring ARIA or of worsening ARIA in patients with more than two episodes is unknown. We recommend permanent discontinuation of treatment after the third episode of ARIA-E to reduce the likelihood of a serious adverse event. In addition, treatment should be discontinued if MRI reveals any macrohemorrhage, more than one area of superficial siderosis, and if the patient has severe symptoms such as seizures. Discontinuation should be considered if MRI demonstrates more than 10 incident microhemorrhages since the start of treatment. Aducanumab should be discontinued if any condition requiring anticoagulation develops (e.g., atrial fibrillation, deep vein thrombosis, pulmonary embolism, hypercoagulable state). When patients progress to moderate-severe dementia stages of AD, continued treatment with aducanumab can be reconsidered and discussed with the patient and care partner. This conversation should be personalized to the context of the patient’s circumstances, clinical status and trajectory perceived meaningfulness of continued treatment, patient and care partner preferences, and uncertainties regarding potential benefit as well as burden and risks. These recommendations addressing the management of recurrent ARIA and when to discontinue treatment are more specific than those of the previous AURs (3).

Detection of subtle ARIA on MRI scans requires a proficient radiologist, and these lesions can go undetected by radiologists not specifically experienced in the detection of ARIA (32). Training programs for radiologists interpreting scans where aducanumab is administered might improve ARIA detection and management(5). Providing centralized resources for review of baseline MRIs and consultation on questions regarding ARIA would provide more access to expertise for ARIA prevention and management. Deploying resources of this type could be considered by those developing monoclonal antibodies.

In the context of rigorous and structured clinical trials and clinician-researchers with advanced knowledge of detection and management of ARIA, the consequences of ARIA were rarely severe enough to meet the definition of serious adverse event (0.3% of trial participants), and management rarely required hospitalization. With the early clinical experience suggesting that serious adverse events may be more common in APOE4 carriers and that the risk is greatest in APOE4 homozygotes, institutions and practices providing aducanumab as a treatment option can engage in anticipatory planning, including a written safety management plan for suspected serious or severe ARIA (Table 4). Management of such patients depends on clinician judgement but might include expeditiously obtaining brain MRI that is read by a radiologist experienced in detection of ARIA if a scan has not already obtained. In cases of severe or serious ARIA-E or ARIA-H (as defined in the trial protocols), monitoring neurologic status closely, including surveillance for potential seizures, and early empiric administration of high dose intravenous corticosteroids to promote accelerated resolution of severe edema should be considered(4). Repeat imaging may be necessary if the patient deteriorates. Such planning will vary by practice setting but might include alerting institutional leaders, intensivists, and clinicians familiar with management of patients with cerebral amyloid angiopathy-related inflammation and other conditions with fulminant brain edema to the possible admittance of cases of severe ARIA.

## Appropriate Discussions with Patients and Families

Shared decision-making and information exchange is a central tenet of best clinical practices. Successful communication is critical for decision support of complex choices requiring understanding of both potential benefits and harms associated with treatment. Patients with MCI and mild AD dementia often have sufficient cognitive capacities to engage in informed discussions and to grasp consequences. Choices characterized by risk and ambiguity are most difficult for these patients (33), and decision supports such as visual aids, repeated discussions, and education of care partners and family members may enhance the patient’s ability to understand and appreciate the treatment and care delivery plan.

Treatment expectations should reflect the characteristics of disease-modifying therapies. The goal of therapy is slowing of loss of function and delay of more advanced disability; patients are not expected to improve (34). The magnitude of the effect is variable, and information is not available to allow prediction of whether a specific individual patient will experience or exhibit benefit.

The EMERGE and ENGAGE trials included very few minority participants and the effect of aducanumab in these patients is unknown. Transparency in communications related to this issue is imperative as minority patients and their families consider therapy with aducanumab.

## Appropriate Practice Adjustments to Accommodate Aducanumab

Aducanumab represents an unprecedented type of therapy and makes new requirements on clinicians and health care systems. Comprehensive clinical assessment, amyloid confirmation, evaluation for exclusionary factors, genotyping as a routine part of care, establishment of schedules for MRI monitoring, managing ARIA when it occurs, communicating treatment expectations, and discussing practical requirements for treatment adherence to patients and families have not previously been obligatory as they are for proper use of aducanumab. Expertise in amyloid PET interpretation or lumbar puncture for CSF confirmation of amyloid abnormalities, MRI interpretation, and treatment infusion are resources that must be readily available.

Practical considerations such as reliable transportation for patients to infusion sessions and arrangements for dependable means of communicating with patients about MRI results if ARIA occurs must be planned. Best practices for patient care include mechanisms for tracking test results, rapid communication of MRI results from radiologist to the treating clinician, scheduling of infusions, and dose adjustments (35).

## Aducanumab and Other Anti-Amyloid Monoclonal Antibodies

### Accelerated Approval

Aducanumab was approved by the FDA using the accelerated approval mechanism available for drugs in the absence of definitively proven clinical efficacy but with effects on a biomarker considered reasonably likely to predict clinical outcomes(2). Accelerated approval is intended to provide earlier access to drugs for serious diseases when there is residual uncertainty at the time of approval regarding the drug’s ultimate clinical benefit. The biomarker on which aducanumab approval was based was lowering of plaque amyloid on amyloid PET(36). The FDA observed that there was a generally consistent association between the reduction in Aβ plaque load and the improvement in Clinical Dementia Rating - Sum of Boxes (CDR-SB) score in the aducanumab clinical trials and that there were similar relationships between Aβ plaque reduction and improvement on clinical outcomes observed with other anti-amyloid monoclonal antibodies in development (37, 38). The accelerated approval mechanism allows for removal of a drug from the market if post-approval studies do not confirm clinical benefit. Post-approval studies will assess the effectiveness of aducanumab.

### Monoclonal Antibodies

Two other anti-amyloid monoclonal antibodies --- lecanemab, donanemab --- will be presented to the FDA for consideration for accelerated approval based on clinical data accompanied by Aβ plaque lowering demonstrated on Aβ PET (37, 38). Another anti-amyloid monoclonal antibody --- gantenerumab --- will complete its clinical development program in 2022 (39). Clinical practice guidance for use of these agents if approved will evolve as experience with them materializes. There are important differences in delivery (intravenous infusion, subcutaneous injection), dosing, and titration among the antibodies. Comparative information regarding efficacy or ARIA rates is not yet available. Antibodies such as solanezumab that target monomeric Aβ peptides rather than larger species do not dramatically reduce amyloid plaque load, are not associated with ARIA, and appear to differ substantially from plaque-lowering antibodies (40). AURs may be valuable for other monoclonal antibodies that are approved for clinical use. Some learnings from aducanumab may be applicable to emerging antibodies while other AURs may be specific to each agent.

### Real-World Use of Aducanumab

Post-approval use of therapies differs from the controlled, monitored determination of efficacy and safety characteristic of clinical trials. Patients receiving treatment in real-world settings may differ substantially from those included in trials (41). Trial entry criteria exclude many of the types of patients seen in real-world practices even when clinical definitions and amyloid confirmation are applied. Trial patients are generally younger, healthier, better educated, are less likely to have comorbidities (which might be associated with adverse effects), and more likely to be White than real-world patients (42). Proximity to a trial center where experienced staff conduct clinical trials also limits the type of patient participating in trials. Trials have centralized safety monitoring that is lacking in the real-world setting. For these reasons, clinicians must be especially vigilant in providing aducanumab to patients in the real-world setting.

## Summary

These updated AURs provide the basis for safe use of aducanumab and describe best practices for integrating aducanumab into the care of patients with MCI or mild dementia due to AD. The AURs stress the need for careful patient selection with confirmation of the presence of brain amyloid and exclusion of patients with vascular changes that may put the patient at risk for ARIA. The AURs emphasize the importance of excluding patients that have medical conditions that may predispose them to ARIA (e.g, history of autoimmune disorders, evidence of cerebral amyloid angiopathy or other cerebrovascular disease) or circumstances that may increase their risk of ARIA complications (e.g, history of seizures). The AURs suggest obtaining MRIs prior to the 5th, 7th, 9th, and 12th infusions to allow early detection of ARIA and to increase the number of scans obtained in the early stages of treatment when ARIA is most likely to occur. APOE genotyping is recommended to facilitate better risk assessment for ARIA and to allow clinicians to have more informed discussions with potential candidates for aducanumab treatment. Emerging data suggest that APOE4 homozygotes are at particularly high risk for ARIA and ARIA complications. Symptomatic or severe ARIA are rare events but preparedness for management of the few cases requiring hospitalization and expert management is necessary. Transparent, patient-centered discussions with potential treatment recipients and families is key to shared-decision making. The rigorous application of patient selection criteria and on-going monitoring characteristic of clinical trials are not typical of clinical practice with an approved treatment. Adherence to the AUR update will facilitate the use of aducanumab in real-world settings with safety similar to that observed in clinical trials.

## Figures and Tables

**Table 1.** Principal new elements of the Updated Appropriate Use Recommendations

• Recommend more emphasis on past medical conditions that may potentially place the patient at increased risk for ARIA or from complications potentially related to ARIA such as evidence of pre-existing autoimmune or inflammatory conditions, history of seizures, transient ischemic attacks, cerebrovascular disease, or extensive white matter changes in the brain
• Recommend APOE genotyping to allow better informed discussions with patients and their care partners concerning the risk for ARIA; these events occur more in patients heterozygous for the APOE4 than in noncarriers and more in those that are homozygous than in heterozygotes. ARIA may potentially be more likely to be recurrent, severe, or serious in individuals who are homozygous for the APOE4 allele.
• Recommend that MRIs be obtained routinely before the 5th, 7th, 9th, and 12th doses of aducanumab.
• Recommend stopping aducanumab therapy for any of the following: ○ Any macrohemorrhage ○ More than 1 area of superficial siderosis ○ More than 10 microhemorrhages occurring since initiation of treatment ○ More than 2 episodes of ARIA ○ Severe symptoms of ARIA ○ Development of any medical condition that requires anticoagulation (e.g., atrial fibrillation, deep vein thrombosis, pulmonary embolism, hypercoagulable state)
• For the most severe symptomatic cases of ARIA, recommend beginning high-dose glucocorticoid therapy; a regimen to be considered is methylprednisolone 1 gm intravenously per day for 5 days followed by oral prednisone, 60 mg per day, slowly tapered over weeks or months.
• For patients with seizures or electroencephalographic evidence of epileptiform activity, recommend treatment with anticonvulsants
• With progression to moderate/severe stages of dementia, recommend reconsideration of aducanumab therapy in the context of the patient’s circumstances, clinical status and trajectory, perceived meaningfulness of continued treatment, patient and care partner preferences, and uncertainties regarding potential benefit as well as burden and risks
• Recommend greater equity of therapeutic opportunity

**Table 2.** Imaging, laboratory, and clinical characterization of patients being considered for treatment with aducanumab

Participant Feature	Appropriate Use in Clinical Practice
Age	50-85; younger or older patients meeting all other criteria for treatment may be considered candidates for aducanumab
Diagnosis	MCI due to AD or mild AD dementia
Cognitive states	Mild decline of cognition with no or limited impairment of activities of daily living established by objective cognitive testing
Amyloid status	Amyloid positive PET (visual read) or CSF findings consistent with AD
Genetic testing	APOE genotype determined
Neurological examination	Non-AD neurological disorders excluded
Cardiovascular history	Stable cardiovascular conditions required
Medical history	Stable medical conditions required; patients with history of autoimmune disorders or seizures excluded
Psychiatric history	Stable psychiatrically
Clotting status	Patients with bleeding disorders or on anticoagulants excluded
Concomitant medications	Patients can be on standard of care with cholinesterase inhibitors and memantine
Laboratory studies	Normal serum vitamin B12 level, thyroid stimulating hormone (TSH), metabolic panel and liver function tests, complete blood count, comprehensive clotting studies and platelet count Normal erythrocyte sedimentaton rate and C-reactive protein
Baseline MRI	None of the following: • Acute or subacute hemorrhage • Macrohemorrhage • Cortical infarction larger than 1.5 cm • One lacunar infarction larger than 1.5 cm • More than four microhemorrhages • More than one area of superficial siderosis • Extensive white matter disease indicative of ischemic injury
Informed consent	Patient and care partner must understand the nature and requirements of therapy (e.g, monthly infusions to be performed indefinitely) and the expected outcome of therapy (removal of amyloid and slowing of decline of clinical features)

AD – Alzheimer’s disease; APOE – apolipoprotein E; MRI – magnetic resonance imaging; PET – positron emission tomography

**Table 3.** Symptoms and signs consistent with ARIA that should trigger consideration of out-of-sequence MRI for patients receiving aducanumab

• Acute or subacute onset of new focal neurological signs or symptoms
• Headache
• Confusion/altered mental status/delirium/disorientation
• Dizziness/vertigo
• Nausea
• Vomiting
• Fatigue
• Blurred vision
• Vision disturbance/impairment
• Gait disturbance
• Seizures

**Table 4.** Patient care can be optimized by development of a triage strategy for evaluation and management of patients with symptoms and signs of severe ARIA. The plan will vary to accommodate clinical judgement as well as institutional resources and circumstances but will typically include these elements

• Referral of patient to emergency department for thorough assessment of suspected/known ARIA
• Brain MRI without contrast enhancement if not already obtained (FLAIR, T2*-GRE or SWI, and DWI sequences)
• MRI review by a reader proficient in detection of ARIA (preferably with access to past MRIs for comparison) and rapid communication between MRI reader and clinicians responsible for patient’s aducanumab treatment and AD care
• Discontinuation of anti-amyloid therapy
• Consultation by a neurologist, preferably a vascular neurologist with experience in management of ARIA-like syndromes
• Admittance to hospital ward for close neurologic monitoring and tiered level of monitoring and management
• Admit or transfer to a stroke care unit or neurological intensive care unit if warranted
• Protocols for, when warranted: ○ Early initiation of treatment with intravenous methylprednisolone 1 g/day for 5 days ○ Conducting electroencephalography to detect epileptiform activity ○ Treatment with anticonvulsants for seizure management or prophylaxis if electroencephalography suggests they are indicated ○ Consideration of additional immunosuppressive treatment if not responding to methylprednisolone after 5 days of treatment ○ Plan transition to oral steroid treatment and taper as outpatient
• Support and communicate with patient and family members/care partners throughout the event with informed patient-centered decision making
